# QMugs, quantum mechanical properties of drug-like molecules

**DOI:** 10.1038/s41597-022-01390-7

**Published:** 2022-06-07

**Authors:** Clemens Isert, Kenneth Atz, José Jiménez-Luna, Gisbert Schneider

**Affiliations:** 1 Department of Chemistry and Applied Biosciences, RETHINK, ETH Zurich, 8093 Zurich, Switzerland; 2 Department of Medicinal Chemistry, Boehringer Ingelheim Pharma GmbH & Co. KG, Birkendorfer Straße 65, 88397 Biberach an der Riss, Germany; 3 ETH Singapore SEC Ltd, 1 CREATE Way, #06-01 CREATE Tower, Singapore, 138602 Singapore

**Keywords:** Cheminformatics, Physical chemistry

## Abstract

Machine learning approaches in drug discovery, as well as in other areas of the chemical sciences, benefit from curated datasets of physical molecular properties. However, there currently is a lack of data collections featuring large bioactive molecules alongside first-principle quantum chemical information. The open-access QMugs (Quantum-Mechanical Properties of Drug-like Molecules) dataset fills this void. The QMugs collection comprises quantum mechanical properties of more than 665 k biologically and pharmacologically relevant molecules extracted from the ChEMBL database, totaling ~2 M conformers. QMugs contains optimized molecular geometries and thermodynamic data obtained via the semi-empirical method GFN2-xTB. Atomic and molecular properties are provided on both the GFN2-xTB and on the density-functional levels of theory (DFT, *ω*B97X-D/def2-SVP). QMugs features molecules of significantly larger size than previously-reported collections and comprises their respective quantum mechanical wave functions, including DFT density and orbital matrices. This dataset is intended to facilitate the development of models that learn from molecular data on different levels of theory while also providing insight into the corresponding relationships between molecular structure and biological activity.

## Abstract (summary)

**Table:** 

Measurement(s)	Quantum Mechanics
Technology Type(s)	density functional theory

## Background & Summary

Machine learning methodologies are increasingly becoming well-established tools in many chemistry-related disciplines, such as drug discovery^1^, material science^2^, and physical chemistry^3^. In recent years, significant progress has been made in quantum-based machine learning (QML) methods^4^, which aim to accurately and computationally inexpensively predict the governing properties of atomistic systems, such as energies and forces^5–12^, dipole moments^13^, wave functions^14,15^ and electron densities^16,17^. Despite the success and promise surrounding the applicability of such approaches, several challenges remain for QML. Arguably, one of the most important challenges is the increasing need for curated, comprehensive datasets^13^. While several options, such as the QM9^18^, ANI-1^19^, or PubChemQC^20^ datasets have paved the way for the development of current-generation QML methods^5–8,21–23^, the computational cost entailed in their generation limits both the scope of the explored chemical space (*e*.*g*., molecule size, atom-type diversity), and prospective modeling applicability^13,24^.

There has been a recent surge in interest in the delta-learning (Δ-learning) of chemical properties, which aims to use a machine learning model to predict a physically relevant quantity, such as those generated by density-functional theory (DFT) by utilizing information extracted with a computationally cheaper method^22,25^ (*e*.*g*., semi-empirical approaches such as GFN2-xTB^26–29^ and PM6^30^). Datasets that enable this type of learning are scarce and could promote the development of accurate models at potentially a fraction of the computational cost of more precise alternatives^31^. Furthermore, datasets that provide three-dimensional conformational data, for a wide variety of chemical space, at levels of theory higher than classical force fields^32,33^, could boost the performance of machine learning methods in predicting properties from ensembles as well as generative models of conformations. Relevant examples include the PubChemQC-PM6^23^ and GEOM^33^ datasets, which include molecules with properties computed using different semi-empirical levels of theory. Finally, there is a clear potential to open up new lines of research by combining biological annotations (*e*.*g*., from molecular databases such as ChEMBL^34^), and additional QM-derived physical information.

**Fig. 1:**
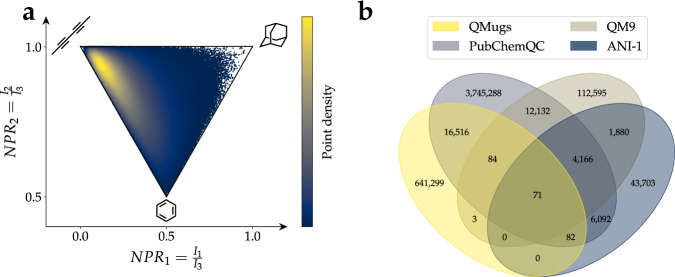
(**a**) Principal-moments-of-inertia plot^39^ for molecules in the QMugs dataset. *NPR*_*x*_ = *x*-th normalized principal moment, *I*_*x*_ = *x*-th smallest principal moment of inertia. (**b**) Venn diagram showing overlap between QMugs and other well-known datasets with DFT-level computed properties: QM9^18^, PubChemQC^20^, and ANI-1^19^. Overlap was computed based on the uniqueness of the InChI representations of the contained molecules. Numbers do not add up to those reported in Table 1 because of InChI strings that occur multiple times.

**Fig. 2:**
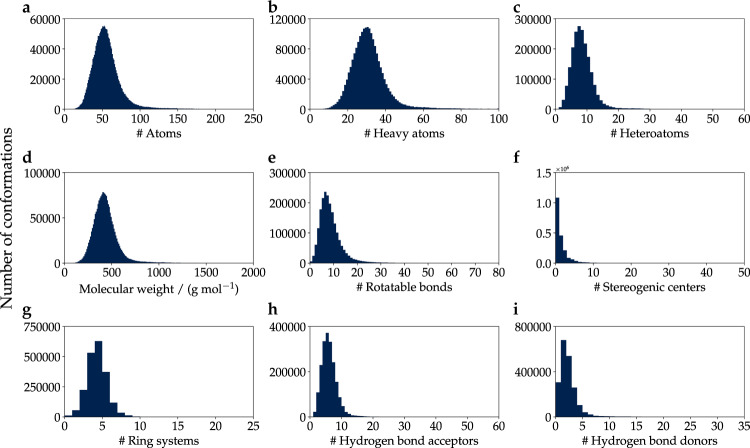
Distribution of properties for the molecules contained in the QMugs dataset.

This work introduces QMugs (**Q**uantum-**M**echanical Properties of Dr**ug**-like Molecule**s**), a data collection of over 665 k curated molecular structures extracted from the ChEMBL database, with accompanying computed quantum mechanical properties. Different levels of theory were combined in these calculations. Per compound, three conformers were generated, and their geometries were optimized using the semi-empirical GFN2-xTB method^26–29^, whereas a comprehensive array of quantum properties was computed at the DFT level of theory using the *ω*B97X-D functional^35^ and the def2-SVP Karlsruhe basis set^36^. The data collection presented herein is put in the context of other sets that also feature DFT-level properties. A descriptive evaluation against the QM9^18^, ANI-1^19^, and PubchemQC^20^ datasets is provided in Fig. 1b, as well as in Table 1. With an average of 30.6 and a maximum of 100 heavy atoms per compound (Table 1 & Fig. 2), QMugs features molecular samples that are considerably larger than those provided by other previously-reported datasets. QMugs also provides a larger number of distinct molecules than QM9 and ANI-1. Though the total number of provided molecules in QMugs is lower than that provided in PubChemQC, QMugs provides multiple conformers per molecule and therefore enables the training of QML models which can differentiate between molecular constitution and conformation. QMugs additionally provides a wide range of properties on multiple levels of theory. Furthermore, the vast majority of the included compounds (~641 k, 96.3%) were previously unreported in other DFT data collections, while also providing equivalent information at additional levels of theory, namely GFN2-xTB. QMugs provides quantum mechanical wave functions represented as local bases of atomic orbitals (*i*.*e*., DFT density and orbital matrices). Single-point properties as well as wave functions were computed with the Psi4 software suite^37^ for all the conformers (~2.0 M) present in the database. As previously reported for the ChEMBL database^38^, most of the considered drug-like molecules in this study fall within the rod-disk axis in the principal moments of inertia plot^39^ (Fig. 1a).

**Table 1:** Descriptive statistics of the dataset reported herein in the context of other DFT-level molecular datasets and the information provided by each.

Dataset	Unique compounds	Total conformations	Heavy atoms max (mean)	Method	Δ-learning possible	Wave functions
QM9	133,885	133,885	9 (8.8)	B3LYP/6–31 G(2df,p)	✗	✗
ANI-1	57,462	22,057,374	8 (7.1)	*ω*B97X/6–31 G(d)	✗	✗
PubChemQC	3,982,436	3,982,436	51 (14.1)	B3LYP/6–31 G(d)	✗	✓
QMugs	665,911	1,992,984	100 (30.6)	GFN2-xTB + *ω*B97X-D/def2-SVP	✓	✓

The number of molecules for PubChemQC corresponds to that available on the website of the project^57^. Heavy atom averages are weighted by the number of conformations.

Overall, the potential utility of the presented dataset is fourfold: (i) it will provide researchers with a dataset containing substantially larger molecules than previously reported, in order to either directly predict quantum chemical properties, or to learn a property mapping between two popular quantum mechanical levels of theory (*i*.*e*., GFN2-xTB and *ω*B97X-D/def2-SVP); (ii) it will facilitate the development of novel machine-learning methodologies for the generation of molecular conformations and molecular property predictions via their ensembles; (iii) it will facilitate the development of novel deep learning frameworks for the prediction of the quantum mechanical wave function in a local basis of atomic orbitals; and (iv) it will enable research towards the exploration of quantum featurization in the context of pharmacologically relevant, annotated biological data.

## Methods

**Fig. 3:**
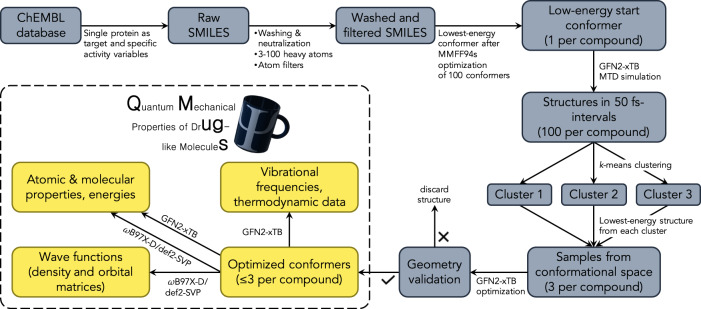
Overview of the data generation process. Molecules were extracted from the ChEMBL database, standardized, and filtered, and starting conformers were generated using the RDKit software package. Metadynamics (MTD) simulations were performed using the GFN2-xTB semi-empirical method to generate three diverse conformations before final geometry optimization. Molecules that did not pass a series of geometric sanity checks were removed. DFT-level properties (*ω*B97X-D/def2-SVP) were computed using Psi4 software.

Molecules were extracted from the ChEMBL database^34^ (version 27). Conformers were generated using RDKit (http://www.rdkit.org) and GFN2-xTB^26–29^. DFT (*ω*B97X-D/def2-SVP) calculations were carried out via Psi4^37^. A similar approach was adopted in a previous study on transition-metal complexes^13^. An overview of the data processing pipeline is given in Fig. 3, while individual steps are described in more detail in the following subsections.

In chemical terminology, the term “conformation” refers to any arrangement of atoms in space, whereas “conformer” refers to a conformation that is a local minimum on the potential energy surface of the molecule^40^. In the analyses that follow, the term “conformation” is loosely used to refer to both, unless explicitly mentioned otherwise.

### Data extraction and SMILES processing

Single-protein targets with assay information for at least 10 compounds with unique internal identifiers were extracted from the ChEMBL database. Several activity and annotation filters were subsequently applied to these compounds (see Supporting Information for a detailed query description). This procedure resulted in 685,917 molecules with unique external identifiers (ChEMBL-IDs), represented by their Simplified Molecular Input Line Entry Specification (SMILES)^41^. Molecules were neutralized, and salts and solvents were removed (“washing”) using the ChEMBL Structure Pipeline package^42^. For compounds consisting of multiple separate fragments, all fragments except the one with the highest number of heavy atoms were discarded after the washing. Additionally, molecules containing fewer than 3 or more than 100 heavy atoms, as well as radical species and molecules with a net charge different from zero after the attempted neutralization, were removed. Atom types included in the QMugs dataset are hydrogen, carbon, nitrogen, oxygen, fluorine, phosphorus, sulfur, chlorine, bromine, and iodine.

### Conformer generation and optimization

With the procedure described herein, a compromise between efficient molecular conformational search and practical computational expense considerations was sought. Similar to previous studies^13,43^, the semiempirical GFN2-xTB method was used as a surrogate for full DFT geometry optimization, as the latter is associated with challenging computational costs when considering the size and number of molecules in the QMugs dataset.

The RDKit (http://www.rdkit.org) implementation of the Experimental-Torsion Knowledge Distance Geometry (ETKDG) method^44^ was used to generate up to 100 conformers for each molecule, with a maximum of 1000 embedding attempts and an initial coordinate assignment using distance-matrix eigenvalues and default settings (boxSizeMult = 2.0, forceTol = 1e-3). Upon no successful conformer generation, it was re-attempted via random assignment of the starting coordinates. The resulting conformers were further minimized using the Merck molecular force field^45^ (MMFF94s) for a maximum of 1000 iterations, with default settings (nonBondedThresh = 100.0). The lowest-energy conformer (according to the selected force field) for each structure was then used as a starting point for meta-dynamics (MTD) simulations. Stereocenters that were previously undefined in the SMILES extraction procedure were assigned in this conformer generation process.

For each generated conformer, an MTD simulation was performed with the xTB software package^28^ for a duration of 5 ps with time steps of 1 fs, at a temperature of 300 K. The biasing root-mean-square deviation (RMSD) potential used for all MTD simulations is given by ${E}_{{\rm{bias}}}^{{\rm{RMSD}}}={\sum }_{i=1}^{N}{k}_{i}\exp \left(\alpha {\Delta }_{i}^{2}\right)$, where *N* is the number of reference structures, *k*_*i*_ the pushing strength, Δ_*i*_ the collective variable (*i*.*e*., the RMSD between structure *i* and a reference structure), and *α* the width of the Gaussian potential used in the RMSD criterion. Simulations were carried out with α = 1.2^−1^ and *k*_*i*_ = 0.2 mEh with snapshots taken every 50 fs, resulting in 100 conformations stored with their corresponding energies. To obtain conformationally diverse samples, these structures were subsequently clustered into three groups via the *k*-means^46^ algorithm, as implemented in the scikit-learn^47^ (version 0.23.1) Python package using the pairwise RMSD of the aligned structures as molecular features. The conformation with the lowest-energy value from each cluster was then selected for further processing. The three resulting conformations for each molecule were then optimized using the GFN2-xTB^26–29^ method using energy and gradient convergence criteria of 5 × 10^−6^ Eh and 1 × 10^−3^ Eh α^−1^, respectively, and the approximate normal coordinate rational function optimizer (ANCopt). Harmonic frequencies, entropies, enthalpies and heat capacities at 298.15 K were extracted at the end of the geometry optimization process. Structures for which vibrational frequencies with imaginary wave numbers were obtained — indicative of failure to reach energy minima — were subjected to additional optimizations until no significant ones remained, up to a maximum of 100 attempts.

### Quantum mechanical calculations

Single-point electronic calculations were performed for the optimized geometries using the *ω*B97X-D quantum functional and the def2-SVP basis set as implemented in the open-source quantum-chemistry software suite Psi4^37^. Single-point properties such as formation and orbital energies, dipole moments, rotational constants, partial charges, bond orders, valence numbers, as well as wave functions including *α* and *β* DFT-density matrices, orbital matrices, and the atomic-orbital-to-symmetry-orbital transformer matrix were obtained. For practical reasons, 52 structures whose DFT calculations required computational resources that exceeded empirically determined limits, or for which calculations were unsuccessful, were discarded (see Supporting Information for details).

## Data Records

All computed molecular structures, as well as their corresponding properties and wave functions are accessible through the ETH Library Collection service^48^.

### Format specification

A summary.csv comma-separated file contains computed molecular-level properties and additional annotations. A compressed tarball file (structures.tar.gz) of ~7 gigabytes (GB) contains plain MDL structure-data files^49^ (SDFs) with embedded atomic and molecular properties, grouped in sub-directories according to their respective ChEMBL identifiers. These SDFs include single-point electronic properties calculated on the GFN2-xTB and *ω*B97X-D/def-SVP levels of theory, as described in Table 2. A second compressed tarball file (vibspectra.tar.gz, ~3 GB) contains vibrational spectra.

**Table 2:** Calculated properties as stored in the SDFs of the QMugs data collection.

Property	Symbol	Unit	Key	Δ-ML
ChEMBL identifier	—	—	CHEMBL_ID	
Conformer identifier	—	—	CONF_ID	
Total energy	*U*_*RT*_	*E*_h_	GFN2:TOTAL_ENERGY	♦
Internal atomic energy	*E*_Atom_	*E*_h_	GFN2:ATOMIC_ENERGY	
Formation energy	*E*_Form_	*E*_h_	GFN2:FORMATION_ENERGY	♦
Total enthalpy	*H*_*RT*_	*E*_h_	GFN2:TOTAL_ENTHALPY	
Total free energy	*G*_*RT*_	*E*_h_	GFN2:TOTAL_FREE_ENERGY	
Dipole (*x*, *y*, *z*, total)	*μ*	D	GFN2:DIPOLE	♦
Quadrupole (*xx*, *xy*, *yy*, *xz*, *yz*, *zz*)	*Q*_*ij*_	D Å	GFN2:QUADRUPOLE	
Rotational constants (*A*, *B*, *C*)	*A*, *B*, *C*	cm^−1^	GFN2:ROT_RONSTANTS	♦
Enthalpy (vib., rot., transl., total)	Δ*H*	cal mol^−1^	GFN2:ENTHALPY	
Heat capacity (vib., rot., transl., total)	*C*_*V*_	cal K^−1^ mol^−1^	GFN2:HEAT_CAPACITY	
Entropy (vib., rot., transl., and total)	Δ*S*	cal K^−1^ mol^−1^	GFN2:ENTROPY	
HOMO energy	*E*_HOMO_	*E*_h_	GFN2:HOMO_ENERGY	♦
LUMO energy	*E*_LUMO_	*E*_h_	GFN2:LUMO_ENERGY	♦
HOMO-LUMO gap	*E*_Gap_	*E*_h_	GFN2:HOMO_LUMO_GAP	♦
Fermi level	*E*_Fermi_	*E*_h_	GFN2:FERMI_LEVEL	
Mulliken partial charges	*δ*_*M*_	*e*	GFN2:MULLIKEN_CHARGES	♦
Covalent coordination number	*N*_coord_	—	GFN2:COVALENT_COORDINATION_NUMBER	
Molecular dispersion coefficient	*C*_6_	a.u.	GFN2:DISPERSION_COEFFICIENT_MOLECULAR	
Atomic dispersion coefficients	*C*_6_	a.u.	GFN2:DISPERSION_COEFFICIENT_ATOMIC	
Molecular polarizability	*α*(0)	a.u.	GFN2:POLARIZABILITY_MOLECULAR	
Atomic polarizabilities	*α*(0)	a.u.	GFN2:POLARIZABILITY_ATOMIC	
Wiberg bond orders	*M*_*AB*_	—	GFN2:WIBERG_BOND_ORDER	♦
Total Wiberg bond orders	${\sum }_{A(A\ne B)}{M}_{AB}$	—	GFN2:TOTAL_WIBERG_BOND_ORDER	♦
Total energy	*U*_*RT*_	*E*_h_	DFT:TOTAL_ENERGY	♦
Total internal atomic energy	*E*_Atom_	*E*_h_	DFT:ATOMIC_ENERGY	
Formation energy	*E*_Form_	*E*_h_	DFT:FORMATION_ENERGY	♦
Electrostatic potential	*V*_ESP_	V	DFT:ESP_AT_NUCLEI	
Löwdin partial charges	*δ*_*L*_	*e*	DFT:LOWDIN_CHARGES	
Mulliken partial charges	*δ*_*M*_	*e*	DFT:MULLIKEN_CHARGES	♦
Rotational constants (*A*, *B*, *C*)	*A*, *B*, *C*	cm^−1^	DFT:ROT_CONSTANTS	♦
Dipole (*x*, *y*, *z*, total)	*μ*	D	DFT:DIPOLE	
Exchange correlation energy	*E*_*XC*_	*E*_h_	DFT:XC_ENERGY	
Nuclear repulsion energy	${\widehat{V}}_{eN}$	*E*_h_	DFT:NUCLEAR_REPULSION_ENERGY	
One-electron energy	${\widehat{T}}_{e}$	*E*_h_	DFT:ONE_ELECTRON_ENERGY	
Two-electron energy	${\widehat{V}}_{ee}$	*E*_h_	DFT:TWO_ELECTRON_ENERGY	
HOMO energy	*E*_HOMO_	*E*_h_	DFT:HOMO_ENERGY	♦
LUMO energy	*E*_LUMO_	*E*_h_	DFT:LUMO_ENERGY	♦
HOMO-LUMO gap	*E*_Gap_	*E*_h_	DFT:HOMO_LUMO_GAP	♦
Mayer bond orders	*M*_*AB*_	—	DFT:MAYER_BOND_ORDER	
Wiberg-Löwdin bond orders	*W*_*AB*_	—	DFT:WIBERG_LOWDIN_BOND_ORDER	♦
Total Mayer bond orders	${\sum }_{A(A\ne B)}{M}_{AB}$	—	DFT:TOTAL_MAYER_BOND_ORDER	
Total Wiberg-Löwdin bond orders	${\sum }_{A(A\ne B)}{W}_{AB}$	—	DFT:TOTAL_WIBERG_LOWDIN_BOND_ORDER	♦

Abbreviations: a.u., atomic units; vib., vibrational; rot., rotational; transl., translational. Properties that enable Δ machine learning are labelled with ♦.

Wave function files (*i*.*e*., DFT density and orbital matrices) as described in Table 3, are split into 100 compressed tarballs (wfns_xx.tar.gz) of ~50 GB each for easier management and downloading. These are supplied as NumPy^50^ (.npy) binary files, which can be read using the Psi4 software package. Molecules (with all conformers grouped together) were assigned at random to the tarballs to enable easy use of subsets of the QMugs dataset without having to download all the files. The assignment of ChEMBL identifiers to tarballs is described in a tarball_assignment.csv file.

**Table 3:** Calculated molecular properties stored in the wave function files provided in the QMugs data collection.

Property	Symbol	Key
Alpha density matrix	*D*_*α*_	matrix, Da
Beta density matrix	*D*_*β*_	matrix, Db
Alpha orbitals	*C*_*α*_	matrix, Ca
Beta orbitals	*C*_*β*_	matrix, Cb
Atomic-orbital-to-symmetry-orbital transformer	*C*_AOTOSO_	matrix, aotoso
Mayer bond orders	*M*_*AB*_	MAYER_INDICES
Wiberg-Löwdin bond orders	*W*_*AB*_	WIBERG_LOWDIN_INDICES

Mayer and Wiberg-Löwdin bond orders included here represent a superset of the bond orders in the SDFs which additionally comprise bond orders for non-covalent bonds.

## Technical Validation

### Optimized geometry sanity checks

Four consecutive geometry checks were performed to filter out structures for which the geometry optimization procedure converged to unrealistic conformations. To determine suitable thresholds for removing a structure from our dataset, the generated geometries were compared to experimental reference values and to DFT-optimized geometries extracted from the PubChemQC dataset^20^. Specifically, we investigated (i) the deviation of bond lengths from experimental reference values, (ii) isomorphism between the initial molecular graphs and those obtained after geometry optimization, (iii) linearity of triple bonds, and (iv) planarity of aromatic rings. Structures were removed from the dataset if they failed any of these tests. In total, 10,986 (0.55%) conformations were discarded from the dataset. Each test is briefly described in the following subsections, with further technical details reported in the Supporting Information.

#### Deviation of bond lengths from experimental reference values

Bond lengths in the optimized structures were compared to average experimental reference values for bonds of the same bond type (single, double, triple, or aromatic) and between the same atoms. Reference values were obtained from the Computational Chemistry Comparison and Benchmark DataBase (CCCBDB)^51^, and the largest absolute bond-length deviation from reference values was recorded per molecule. Bonds for which no reference value was available (0.75%) were omitted. The same analysis was carried out for molecules from the PubChemQC dataset containing the same atom types as QMugs, in order to obtain a comparable set with respect to the present atom types. The PubChemQC set (3,834,382 conformations with reference bond lengths) showed a deviation of 0.06 ± 0.04Å (median ± 1 standard deviation), whereas the QMugs dataset (2,004,003 conformations with reference bond lengths) showed a deviation of 0.07 ± 0.03Å. Based on the observed distribution of bond-length deviations from experimental reference values (Figure S1) and manual investigation of example structures, 0.2 was determined to be a suitable threshold for a conformation to be removed from the dataset, which included 6,131 (0.31%) examples.

#### Molecular graph isomorphism

It was investigated whether atom connectivity could be reconstructed after removing bond information from the generated SDFs. To this end, molecular graphs constructed exclusively from atom positions and types were compared to those obtained using the original atom connectivity (see Supporting Information for details). 1,568 (0.08%) conformations for which the resulting molecular graphs were non-isomorphic failed this test.

#### Deviation of triple bonds from linear geometry

The deviation of triple bonds from their ideal linear geometry was examined. In this investigation, ring triple bonds were not considered owing to routinely-occurring deviations from linear geometry in systems with high ring strain^52^. The largest deviation from a 180 (linear) bond angle was recorded for each molecule containing at least one non-ring triple bond. The same analysis was performed on the PubChemQC dataset^20^. Triple-bond-containing molecules from PubchemQC and QMugs (273,320 and 165,101 samples, respectively) showed deviations of 1.38 ± 1.46° (median ± 1 standard deviation), and 1.46 ± 2.13°, respectively. Based on the observed distribution of triple bond angles from a linear geometry (Figure S2) and manual inspection of structures, a 10° deviation was identified as a suitable threshold. 1,147 (0.06%) conformations failed this test.

#### Deviation of aromatic rings from planar geometry

The planarity of carbon-containing aromatic rings was also investigated. For each molecule containing aromatic carbon atoms, the largest dihedral angle between the two planes spanned by each aromatic carbon atom and its three neighbors was recorded (see Supporting Information for details). The same analysis was performed on the PubChemQC dataset^20^. Molecules from PubchemQC and QMugs (2,391,589 and 1,950,929 conformations with aromatic carbons, respectively) showed median dihedral angles ( ± 1 standard deviation) of 1.70 ± 1.85° and 2.99 ± 2.20°, respectively. Based on the observed distribution of dihedral angles from planar geometries (Figure S3) and manual inspection of structures, 2,769 (0.14%) conformations with aromatic carbon dihedral angles above 15° were discarded.

### Further geometrical assessment

**Fig. 4:**
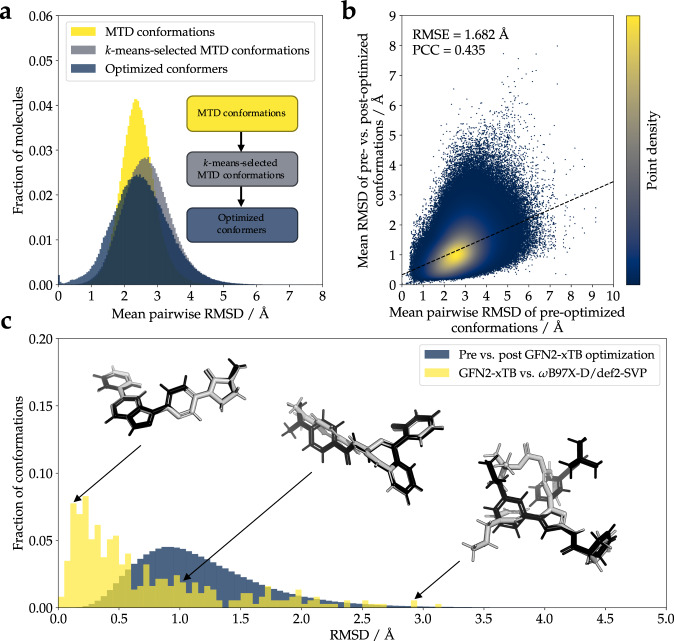
(**a**) Distributions of mean pairwise RMSD of atom positions between conformations of each molecule in the QMugs dataset at different stages along the pipeline. While the *k*-means sampling process selects conformations that are, on average, more geometrically diverse than the average pair of structures generated by MTD simulations, geometry optimization reduces the geometrical diversity between the optimized conformers. (**b**) Change in atom positions during geometry optimization vs. mean pairwise RMSD of conformations before optimization. Molecules with initially more diverse conformations displayed a greater change in atom positions than those with initially less diverse conformations. (**c**) Distribution of RMSD of structures prior to and after optimization with the semi-empirical GFN2-xTB method, and of structures optimized with the same approach vs. with *ω*B97X-D/def2-SVP. The structures of three molecules with varying differences between the two methods are shown as illustrative examples (black and gray correspond to GFN2-xTB and *ω*B97X-D/def2-SVP-optimized structures, respectively). For illustrative purposes, the example molecules are aligned on their substructures.

The changes in the molecular geometries along the applied pipeline were examined in order to evaluate the effects of the applied steps. Figure 4a shows the mean pairwise RMSD of atom positions between the conformations of each molecule at different steps along the pipeline. Conformations sampled during MTD simulations showed a mean pairwise RMSD of 2.40 ± 0.52° (median ± 1 standard deviation). The *k*-means clustering procedure accomplished the envisaged task of sampling conformations with higher geometric diversity (2.67 ± 0.74°). During the geometry optimization process, conformational diversity decreased (2.48 ± 0.86°). Unsurprisingly, for some molecules featuring rigid structures, conformations tended to converge toward the same energy minimum (0.09% of molecules show a mean pairwise RMSD < 0.01° between their optimized conformers).

The degree to which the molecular geometries changed during the final optimization step was further analyzed. Molecules with initially more diverse conformations (higher mean pairwise RMSD of pre-optimized conformations) were shown to undergo a greater chance in atom positions (mean RMSD of pre- vs. post-optimized conformations) during optimization with the GFN2-xTB method (Fig. 4b). The observed heteroscedastic behavior of these two properties indicates that while the mean RMSD of pre- vs. post-optimized conformations tends to increase with higher mean pairwise RMSD of pre-optimized conformations, its variance also increases.

Finally, the suitability of GFN2-xTB as a lower-cost surrogate for DFT-level geometry optimization (Fig. 4c) was confirmed. 500 randomly-chosen structures prior to semi-empirical geometry optimization from the QMugs dataset were further subjected to DFT-level geometry optimization (*ω*B97X-D/def2-SVP), discarding structures that could not be converged in 100 iterations or with the computational resources described in the Supporting Information. The RMSDs between the structures independently optimized at both levels of theory were then measured. The pairs of structures showed RMSDs of 0.47 ± 0.63° (median ± 1 standard deviation), indicating that the chosen semi-empirical method obtains similar geometries to those obtained with more expensive first-principle calculations. Large RMSDs in some example pairs (Fig. 4c) could be interpreted as indicative of convergence to distinct local minima.

For 2,067 molecules, their individual conformations have different SMILES describing two different (*E*)*/*(*Z*) isomers. Those structures are either *α*-*β*-unsaturated ketones, *α*-*β*-unsaturated nitriles, imine, or azo compounds, for which isomerization might be plausible^53,54^. In part due to the applied “washing” procedure, 17,176 molecules can be represented with a SMILES string that is shared with at least one other ChEMBL-ID.

### Validation of single-point properties

**Fig. 5:**
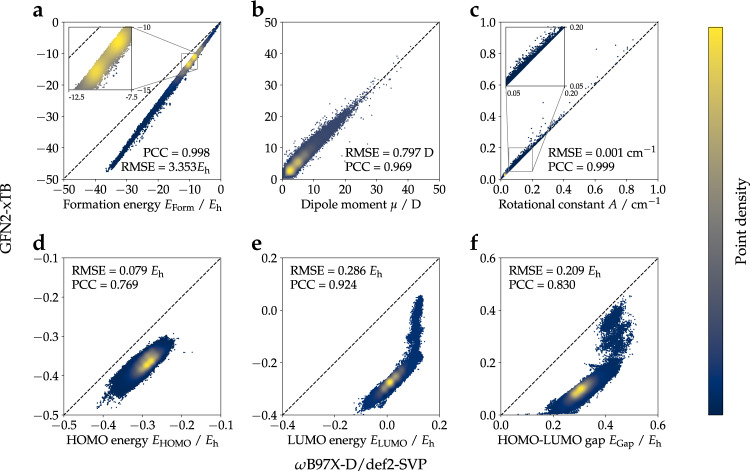
Comparison of molecular properties computed at the two levels of theory considered herein (GFN2-xTB, *ω*B97X-D/def2-SVP) for the molecules contained in QMugs. The molecular formation energy *E*_Form_ *E*_Form_ in (**a**) was calculated by subtracting the atomic *U*_Atom_ contributions from the total molecular energies *U*_*RT*_. Only the rotational constants *A* are shown in (**c**) as their *B* and *C* counterparts showed highly similar values. 22 conformations of small molecules show very large rotational constants and are not shown. RMSE and PCC for rotational constant *A* are 845.834 cm^−1^ and 0.091 respectively, if those structures are included. Abbreviations: RMSE, root mean squared error; PCC, Pearson’s correlation coefficient.

**Fig. 6:**
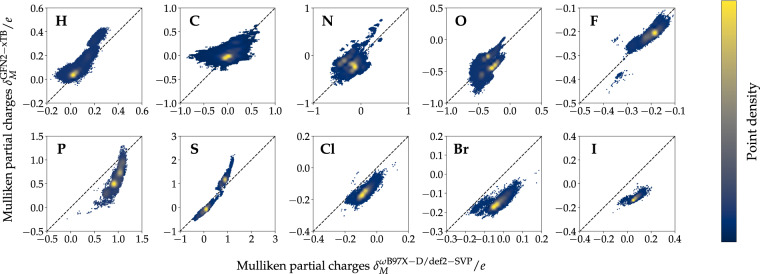
Atom-type-specific partial charge correlations (GFN2-xTB, *ω*B97X-D/def2-SVP) for the QMugs dataset (see Table S1 in the Supporting Information for additional metrics).

**Fig. 7:**
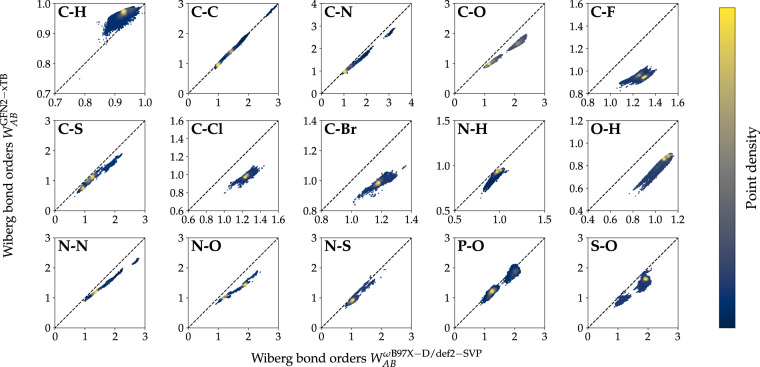
Comparison of Wiberg bond orders between GFN2-xTB and *ω*B97X-D/def2-SVP for the 15 most frequently occurring bond types in the QMugs dataset. The latter level of theory uses Löwdin-orthogonalization. See Table S2 in the Supporting Information for additional metrics. For bond types which occurred > 1 M times in the dataset, a randomly chosen sample of 1 M bonds is plotted.

To validate the general agreement between the two methods employed in this work, the correlation between a series of single-point properties computed on both levels of theory was analyzed. Both global molecular (Fig. 5) and local atomic/bond properties (Fig. 6, 7) were considered. All single-point molecular properties showed a high degree of correlation. Formation energies *E*_Form_ (Fig. 5a), which were obtained by subtracting atomic energies *E*_Atom_ from total internal energies *U*_*RT*_, show a Pearson correlation coefficient (PCC) of 0.998. Dipole moments *μ* and rotational constants *A* (excl. 22 small structures with very high rotational constants; Fig. 5b,c) displayed PCCs of 0.969 and 0.999, respectively. Orbital energies, namely the energies for highest occupied (HOMO) *E*_HOMO_ and lowest unoccupied molecular orbitals (LUMO) *E*_LUMO_ and HOMO-LUMO gap energies *E*_Gap_ showed PCCs of 0.769, 0.924 and 0.830, respectively (Fig. 5d–f). The observed PCCs for all six single-point molecular properties indicate good agreement between the two methods. Atom-type-specific partial charges for the 10 atom-types in QMugs (Fig. 6, Table S1) as well as the 15 most abundant covalent bond types (Fig. 7, Table S2) also showed high correlations between the two methods used herein. Regarding partial charges, 7 out of the 10 atom types considered in QMugs were observed to have PCCs > 0.8, with the remaining carbon, nitrogen, and oxygen atom-types resulting in lower PCCs of 0.574, 0.124, and 0.274, respectively. Regarding bond orders, 10 out of the 15 show PCCs > 0.9 and 14 out of 15 displayed PCCs > 0.75 (see Table S2 for additional metrics). Notably only carbon-fluorine bonds displayed a larger discrepancy between both levels of theory, with an observed PCC of 0.153. The observed correlations in both molecular and atomic single-point properties between GFN2-xTB and *ω*B97X-D/def2-SVP confirm the suitability of the former method as a computationally affordable starting point for Δ-learning of DFT-level properties.

## Usage Notes

All data files can be accessed via any modern web browser, and can be programmatically downloaded using the provided instructions in the archive’s readme. The provided SDFs can be processed using standard cheminformatics software (*for example*, RDKit, KNIME^55^), and wave function files using the Psi4^37^ software package or directly using Numpy^50^.

## Supplementary information

Supplementary Information

## Data Availability

All analyses were supported by the Python programming language (version 3.7.7) and its scientific software stack^50^. Molecular conformations were generated using RDKit (http://www.rdkit.org, version 2020.03.3) and GFN2-xTB^26–29^ (version 6.3.1). All quantum mechanical calculations were carried out with Psi4^37^ (version 1.3.2). Molecular structure visualizations were created using PyMol^56^ (version 2.3.5) and ChemDraw (version 19.1.1.32). The rclone (https://rclone.org, version 1.54.0) WebDAV client was used for all data uploading purposes.
