# Supplementary material for: QMugs, quantum mechanical properties of drug-like molecules
Source: Sci Data. 2022 Jun 7;9:273. doi: 10.1038/s41597-022-01390-7 (PMC9174255; doi:10.1038/s41597-022-01390-7)
Supplement: Supplementary file 1 — Supplementary Information [file 41597_2022_1390_MOESM1_ESM.pdf]

QMugs: Quantum Mechanical Properties of Drug-like Molecules
Supporting Information

Clemens Isert[1,†], Kenneth Atz[1,†], José Jiménez-Luna[1,2,*], and Gisbert Schneider[1,3,*]

[1]Department of Chemistry and Applied Biosciences, RETHINK, ETH Zurich, 8093 Zurich, Switzerland.

[2]Department of Medicinal Chemistry, Boehringer Ingelheim Pharma GmbH & Co. KG, Birkendorfer Straße 65, 88397 Biberach an der Riss, Germany.

[3]ETH Singapore SEC Ltd, 1 CREATE Way, #06-01 CREATE Tower, Singapore 138602, Singapore.

[*]corresponding authors: Gisbert Schneider (gisbert@ethz.ch), José Jiménez-Luna (jose.jimenez@rethink.ethz.ch)

[†]these authors contributed equally to this work

Contents

1 Overlap with other datasets 5
2 ChEMBL SQL query 5
2.1 Extraction of targets 5
2.2 Extraction of compounds 5
3 SMILES filtering 5
4 Computational resources 6
5 Optimized geometry sanity checks 6
5.1 Deviation of bond lengths from experimental reference values 6
5.2 Molecular graph isomorphism 7
5.3 Deviation of triple bonds from linear geometry 7
5.4 Deviation of aromatic rings from planar geometry 8
6 Independent terms of the Schrödinger equation 10
7 Thermodynamic properties 11
8 Additional figures and tables 11

List of Tables

S1 Atom-type-specific atomic partial charge comparisons for the two levels of theory (GFN2-xTB, ωB97X-D/def2-SVP) for the QMugs database. Abbreviations: RMSE, root mean squared error; PCC, Pearson's correlation coefficient. 11
S2 Wiberg bond order comparisons for the two levels of theory (GFN2-xTB, ωB97X-D/def2-SVP) and the 15 most frequent pair-wise atomic covalent bonds in QMugs. Abbreviations: RMSE, root mean squared error; PCC, Pearson's correlation coefficient. 12

List of Figures

S1 *(Top)* Distribution of largest absolute bond-length deviation from experimental reference values per conformation (histogram bin size 5×10^{-4} Å). PubChemQC (3,834,382 conformations with reference bond lengths) shows a deviation of 0.0580 ± 0.0419 Å (median ± 1 standard deviation), whereas QMugs (2,004,003 conformations with reference bond lengths) exhibits a deviation of 0.0687 ± 0.0317 Å. 8,492 (0.22%) and 926 (0.05%) conformations in the PubChemQC and QMugs sets, respectively, have higher deviations than 0.30 Å and are not shown. *(Bottom)* Distribution of the total number of atoms per conformation in both datasets (histogram bin size 1), showing that molecules in the QMugs sample are significantly larger on average. 765 (0.04%) conformations in the QMugs dataset have more than 200 atoms and are not shown. Potentially-arising greater steric clashes in larger molecules may contribute to the slightly higher bond length deviations in the QMugs dataset, compared to the PubChemQC dataset. 7
S2 Distribution of triple bond angle deviations from an ideal 180° angle (histogram bin size 0.05°). Triple bond-containing conformations from PubchemQC (273,320 conformations) and QMugs (165,101 conformations) show a deviation of 1.38 ± 1.46° (median ± 1 standard deviation), and 1.46 ± 2.13°, respectively. 104 conformations (0.04%) in the PubChemQC and 179 molecules (0.11%) in the QMugs sample with higher deviations than 14° are not shown. 8
S3 Distribution of dihedral angle around aromatic carbons (histogram bin size 0.05°). Molecules with aromatic carbons from PubchemQC (2,391,589 conformations) and QMugs (1,950,929 conformations) show a deviation of 1.70 ± 1.85° (median ± 1 standard deviation) and 2.99 ± 2.20°, respectively. 1050 (0.04%) molecules in the PubChemQC dataset and 564 (0.03%) molecules in the QMugs dataset with deviations greater than 20° are not shown. 9
S4 Terms of the molecular Hamiltonian $\hat{H}$ calculated on the ωB97X-D/def2-SVP level-of-theory for molecules in QMugs. (**A**) Nuclear repulsion energy $\hat{V}_{NN}$ in E_H. (**B**) Exchange correlation energy $\hat{V}_{eN}$ in E_H. (**C**) One electron energy $\hat{T}_e$ in E_H. (**D**) Two electron energy $\hat{V}_{ee}$ in E_H. 10
S5 Thermodynamic property distribution for molecules in QMugs calculated on the GFN2-xTB level-of-theory. (**A**) Gibbs free energy G in eV. (**B**) Enthalpy H in eV. (**C**) Fermi level E_{Fermi} in eV. (**D**) Heat capacity C_{Tot}^{Temp} / eV K^{-1} mol^{-1}. (**E**) Entropy S_{Tot}^{Temp} in eV K^{-1} mol^{-1}. (**F**) Partition function enthalpy H_{Tot}^{Temp} / eV mol^{-1}. 11

1 Overlap with other datasets

The overlap between the compounds included in the QMugs dataset with those included in other datasets featuring DFT-properties, namely QM9[1], PubchemQC[2] and ANI-1[3], was investigated. Overlap was computed based on the respective compounds' InChI[4] strings. For each dataset, the molecules were converted from their original formats (QMugs: `.sdf`, QM9: `.xyz`, PubChemQC: `.mol`, ANI-1: SMILES[5], as obtained with the `pyanitools` module included in the ANI-1 repository) to InChIs using Openbabel[6,7] (version 3.1.1). Molecules that could not be successfully converted were skipped. The Venn diagram (Fig. 4, main article) was constructed using the `pyvenn`[8] package. Note that due to different washing procedures, the same compound identifier may refer to different InChI strings in different datasets (*e.g.*, protonated and unprotonated).

2 ChEMBL SQL query

A locally-downloaded MySQL instance of the ChEMBL27 database[9] (version 8.0.19) was queried[10]. Our data extraction procedure is performed in two steps: First, a list of biological targets was extracted. Second, compounds for which a specific activity towards any of these targets was annotated were extracted. Both steps are described in the following.

2.1 Extraction of targets

"Single-protein" targets were selected, for which `activity.standard_types` were reported either as IC_{50}, EC_{50}, K_i, K, K_b, K_a, K_d, K_e, or K_m and in units of nM, or as a set of other `activity.standard_types` (*e.g.*, $-\log K$) and without units. The following types of annotations were excluded: Annotations with an `assay.relationship_type` other than "homologous" or "direct", annotations with an `assay.confidence_score` below 7, annotations with assay descriptions for mutant species, annotations for potential activity duplicates, annotations with assay data validity comments other than "Manually validated" or "null", and annotations with `activity.activity_comments` indicating missing data such as "Not Determined" or "Not tested". Only targets which had annotations for 10 or more unique compounds (as identified by their `activity.molregno` identifier) after this data extraction process were kept.

2.2 Extraction of compounds

For each of the targets extracted in the previous steps, we queried the SQL database for activity annotations. Annotations for which the `activity.standard_value` or the `activity.standard_unit` was missing were discarded. All `activity.standard_values` for annotations were converted to negative decadic logarithm scales (pX) and values below -3 on this scale were corrected to their absolute value. The `activity.standard_values` for annotations with activity comments denoting inactivity were set to 3 on this log-scale. Annotations where the converted `activity.standard_value` lay outside the range of 3-12 were excluded. For each of the compounds that remained, we extracted the ChEMBL-ID and the canonical SMILES.

3 SMILES filtering

The molecules extracted from the ChEMBL database were filtered to exclude the following SMARTS patterns using RDKit[11] (version 2020.03.3.0):

```
B, [*]~P(~[*])(~[*])(~[*])~[*], [*]~S(~[*])(~[*])(~[*])~[*], [S+^3], [P+^3],
[*]~[F,Cl,Br,I]~[*], [Be], [Na], [Al], [Si], [Sc], [Ti], [V], [Cr], [Mn], [Fe], [Co], [Ni], [Cu],
[Zn], [Ga], [Ge], [As], [Se], [Rb], [Sr], [Y], [Zr], [Nb], [Mo], [Tc], [Ru], [Rh], [Pd], [Ag], [Cd],
[In], [Sn], [Sb], [Te], [Cs], [Ba], [La], [Ce], [Pr], [Nd], [Pm], [Sm], [Eu], [Gd], [Tb], [Dy], [Ho],
```

[Er], [Tm], [Yb], [Lu], [Hf], [Ta], [W], [Re], [Os], [Ir], [Pt], [Au], [Hg], [Pb], [Bi], [Fr]

Compounds containing these substructures were excluded as they were either incompatible with the used MMFF94s force field[12] (*e.g.*, B), contained halogens bound to two neighbors (*e.g.*, [*]~[F,Cl,Br,I]~[*]), or elements with reduced relevance for drug-like molecules.

4 Computational resources

For practical reasons, molecules whose DFT-calculations demanded computational resources that exceeded empirically determined limits, were discarded. These limits include a maximum processing power of 4 CPU cores for 24 h wall-time, up to 40 GB of system memory, and 100 GB of scratch space. For the single-point property calculations, this was the case for 9 structures. Additionally, 19 structures (GFN2-xTB) and 24 structures (DFT) were discarded as the respective calculations could not be completed successfully.

5 Optimized geometry sanity checks

We performed four consecutive geometry checks (Sections 5.1 – 5.4) to filter out structures for which geometry optimization had converged to unrealistic conformations. Structures failing any of these tests were removed from the dataset. Note that stated numbers of investigated molecules for the QMugs dataset are larger than the size of the final dataset, as only molecules that passed all geometry checks were included in the final dataset. Note that the reported numbers of conformations failing the tests described in the following subsections do not add up to 10,986 (the number of conformations removed from the dataset) since some conformations failed in multiple tests.

5.1 Deviation of bond lengths from experimental reference values

We searched for bond-length outliers in the optimized structures. As reference values, average bond-length values between atom types from "Standard Reference Database, Computational Chemistry Comparison and Benchmark DataBase"[13] were extracted. For each bond in the optimized structures, the absolute deviation from its respective reference value was calculated. For each conformation, the largest absolute deviation was recorded. If no reference value for a specific bond type between two atom types was found, the bond was not considered for further analysis. This was the case for 876,113 (0.75%) of all bonds in the investigated molecules from the QMugs dataset.
The same procedure was carried out on molecules from the PubChemQC dataset[2] containing the same atom types as the QMugs dataset (Figure S1). The restriction of atom types was made in order to rule out effects of atom types on bond length deviations. This restriction removed 65,316 (1.64%) molecules from the assessment. Further 82,708 (2.08%) molecules which could not be successfully read with RDKit[11] were ignored. Based on the distribution of bond-length deviations from experimental reference values, we discarded molecules above the 0.2 Å threshold. Based on manual inspection of structures at different bond length deviation values, this threshold appeared as a suitable, conservative threshold. 6,131 (0.31%) conformations did not pass this test.

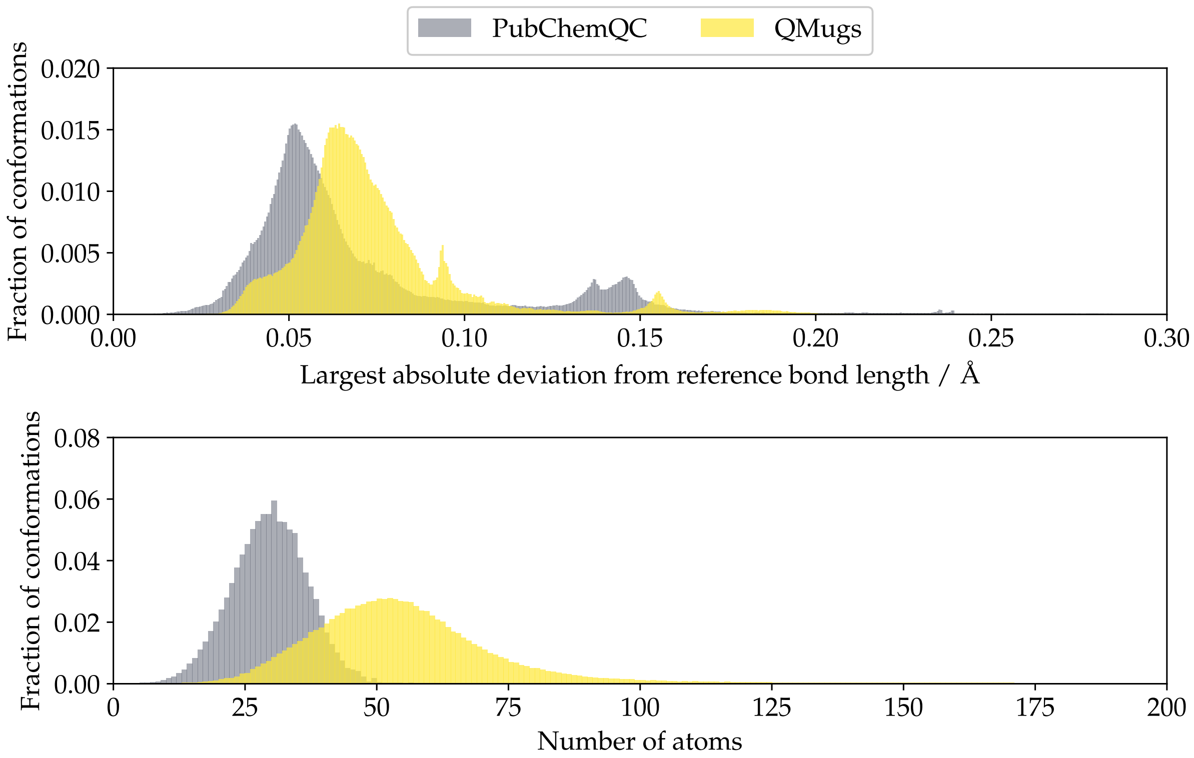

Figure S1: *(Top)* Distribution of largest absolute bond-length deviation from experimental reference values per conformation (histogram bin size 5×10^{-4} Å). PubChemQC (3,834,382 conformations with reference bond lengths) shows a deviation of 0.0580 ± 0.0419 Å (median $\pm$ 1 standard deviation), whereas QMugs (2,004,003 conformations with reference bond lengths) exhibits a deviation of 0.0687 ± 0.0317 Å. 8,492 (0.22%) and 926 (0.05%) conformations in the PubChemQC and QMugs sets, respectively, have higher deviations than 0.30 Å and are not shown. *(Bottom)* Distribution of the total number of atoms per conformation in both datasets (histogram bin size 1), showing that molecules in the QMugs sample are significantly larger on average. 765 (0.04%) conformations in the QMugs dataset have more than 200 atoms and are not shown. Potentially-arising greater steric clashes in larger molecules may contribute to the slightly higher bond length deviations in the QMugs dataset, compared to the PubChemQC dataset.

5.2 Molecular graph isomorphism

We investigated whether heavy-atom connectivity can be reconstructed when removing all bond information from the generated structure-data files (SDF) in the database. SDFs were converted to the .xyz file format (which does not contain bond information) using OpenBabel[6,7] (version 3.1.1). We then attempted to perform a conversion from .xyz to InChI[4], or to SMILES[5] upon failure of the former. If both were unsuccessful, we considered the graph isomorphism check as failed. If either succeeded, however, we compared the molecular graph of generated molecular strings (as read by RDKit) to the one originally obtained from the SDF (which includes bond information). The isomorphism of the molecular graphs was then checked using the NetworkX[14] Python package (version 2.5), considering nodes (representing atoms) in each graph labelled with their respective atom types. We did not use bond types in the previous comparison due to observed high false negative rates related to mislabelled bonds in nitro and other functional groups with multiple resonance structures, among further reasons. 1,568 (0.08%) conformations failed this test.

5.3 Deviation of triple bonds from linear geometry

For each molecule containing a triple bond which is not part of a ring, the deviation of bond angles γ from the ideal 180° (linear) geometry, denoted as $\Delta\gamma$, was assessed. For each non-terminal atom in a non-ring triple bond, the angle between the bonds to its two neighbors was computed. The largest deviation $\Delta\gamma$ from a perfectly linear triple bond was recorded per molecule. We limit this investigation to triple

bonds outside a ring as triple bonds in rings can show substantial deviations from a linear geometry due to high ring strain (*e.g.*, cyclooctyne, the smallest stable, cyclic hydrocarbon accommodating a triple bond, deviates by $\Delta\gamma = 17°$ from a linear geometry[15]). For reference, we performed the same study on all molecules from the PubChemQC dataset[2] which include at least one non-ring triple bond (Figure S2), skipping molecules with could not be read with RDKit. From visual inspection of the distribution of triple bond angle deviations and manual inspection of example structures at different deviation levels, we decided to discard structures with a triple bond angle deviation of $\Delta\gamma > 10°$. 1,147 (0.06%) conformations failed this test. Structures for which GFN2-xTB[16,17,18,19] still indicated significant negative wavenumbers after 100 iterations (17,502 conformations, 0.88%) are denoted in the `summary.csv` file.

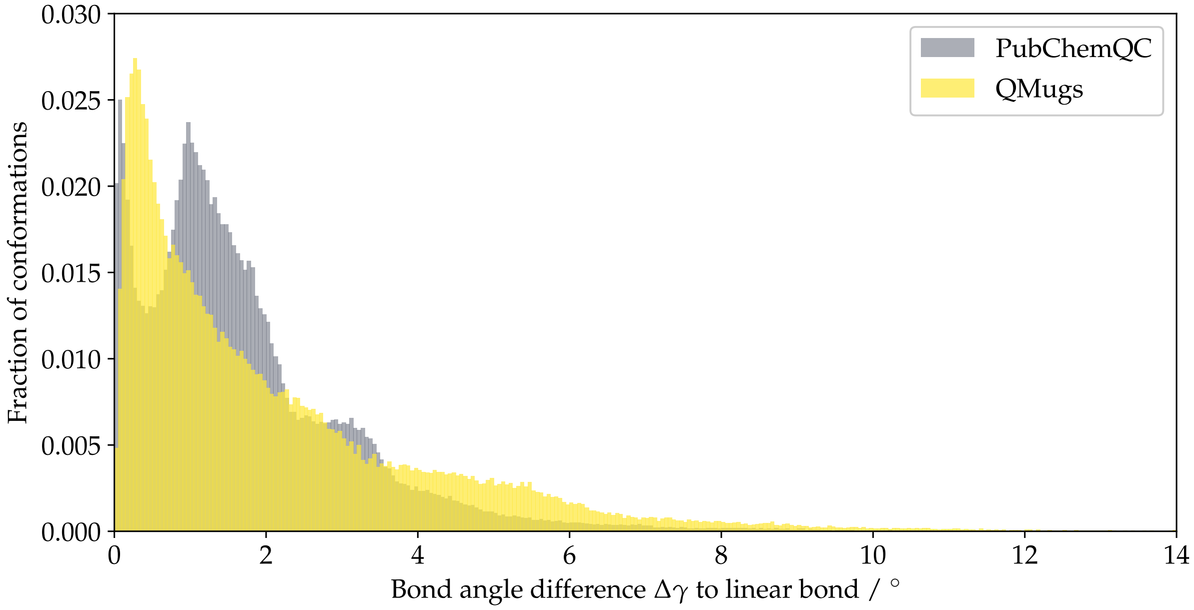

Figure S2: Distribution of triple bond angle deviations from an ideal 180° angle (histogram bin size 0.05°). Triple bond-containing conformations from PubchemQC (273,320 conformations) and QMugs (165,101 conformations) show a deviation of 1.38 ± 1.46° (median ± 1 standard deviation), and 1.46 ± 2.13°, respectively. 104 conformations (0.04%) in the PubChemQC and 179 molecules (0.11%) in the QMugs sample with higher deviations than 14° are not shown.

5.4 Deviation of aromatic rings from planar geometry

We furthermore investigated the planarity of carbon-containing aromatic rings. To do so, we assessed the dihedral angle between the two planes spanned by each aromatic carbon atom and its three neighbors. Angles greater than 90° were corrected to 180°-<angle> to remove directional dependency. Calculations were performed for all order permutations of the aromatic carbon atom and its three neighbors. The largest dihedral angle (and hence the largest deviation from a perfectly planar aromatic ring) was recorded per conformation. We performed the same study on the molecules from the PubChemQC dataset[2] described in Section 5.1 (Figure S3). From visual inspection of the distribution of aromatic carbon dihedral angle deviations and manual inspection of example structures at different deviation levels, we decided to discard structures with an aromatic carbon dihedral angle deviation of 15° or more. 2,769 (0.14%) conformations failed this test.

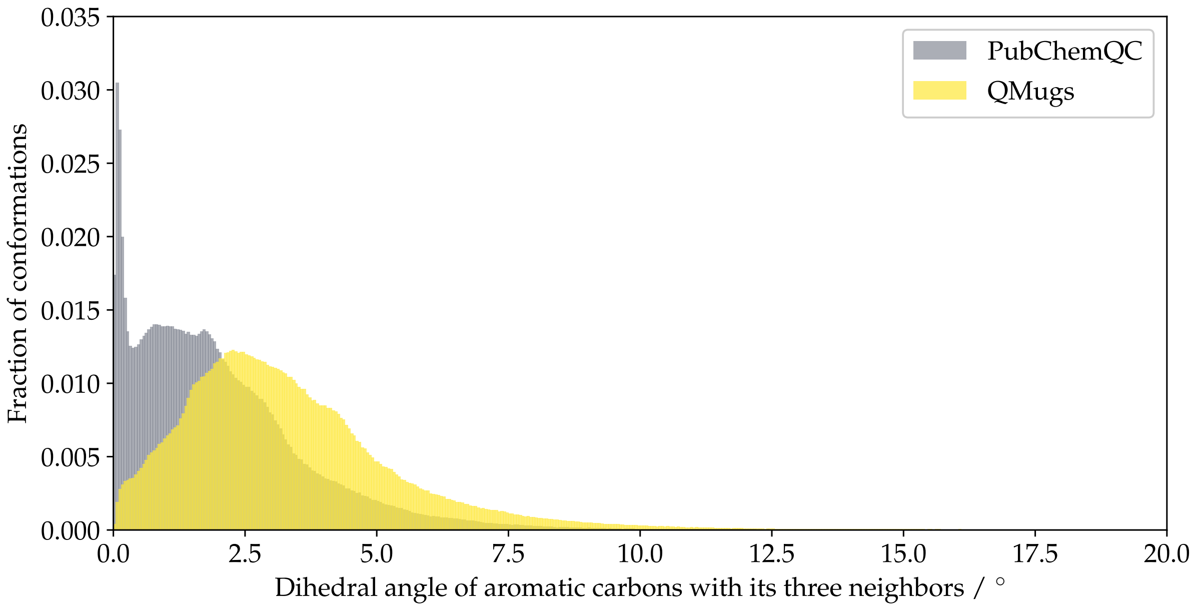

Figure S3: Distribution of dihedral angle around aromatic carbons (histogram bin size 0.05°). Molecules with aromatic carbons from PubchemQC (2, 391, 589 conformations) and QMugs (1, 950, 929 conformations) show a deviation of 1.70 ± 1.85° (median ± 1 standard deviation) and 2.99 ± 2.20°, respectively. 1050 (0.04%) molecules in the PubChemQC dataset and 564 (0.03%) molecules in the QMugs dataset with deviations greater than 20° are not shown.

6 Independent terms of the Schrödinger equation

The Born Oppenheimer approximation[20] defines the molecular energy of the electronic Schrödinger equation as a sum of four independent terms, namely (i) nuclear repulsion energy $\hat{V}_{NN}$, (ii) exchange correlation energy $\hat{V}_{eN}$, (iii) kinetic electron energy also known as one electron energy $\hat{T}_e$, and (iv) electron repulsion energy also known as two electron energy $\hat{V}_{ee}$. The distribution of the four terms in QMugs are visualized in Figue S4.

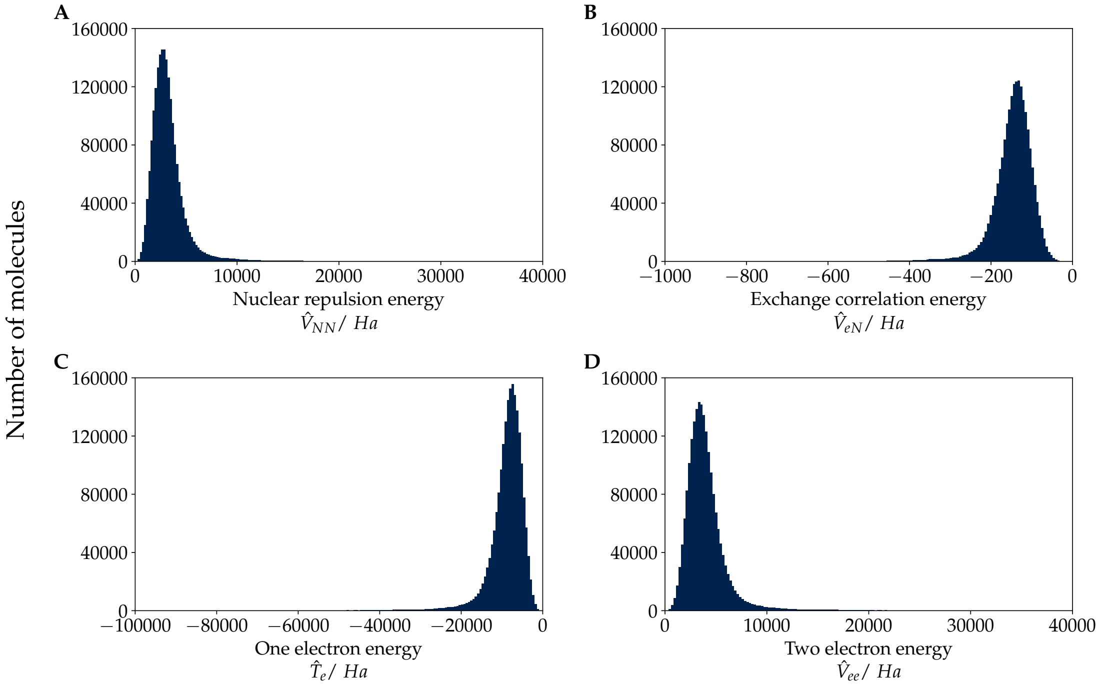

Figure S4: Terms of the molecular Hamiltonian $\hat{H}$ calculated on the ωB97X-D/def2-SVP level-of-theory for molecules in QMugs. (**A**) Nuclear repulsion energy $\hat{V}_{NN}$ in E_H. (**B**) Exchange correlation energy $\hat{V}_{eN}$ in E_H. (**C**) One electron energy $\hat{T}_e$ in E_H. (**D**) Two electron energy $\hat{V}_{ee}$ in E_H.

7 Thermodynamic properties

Thermodynamic properties have been calculated for molecules in QMugs on the GFN2-xTB level-of-theory. Properties include total Gibbs free enegery G, total enthalpy H, Fermi level E_{Fermi}, total heat capacity C_{Tot}^{Temp}, temperature dependent entropy S_{Tot}^{Temp} and enthalpy H_{Tot}^{Temp}. C_{Tot}^{Temp}, S_{Tot}^{Temp} and H_{Tot}^{Temp} correspond to the sum of their individual rotational, transnational and vibrational terms, which can all be found in the structure data files. Their distribution is shown in Figure S5.

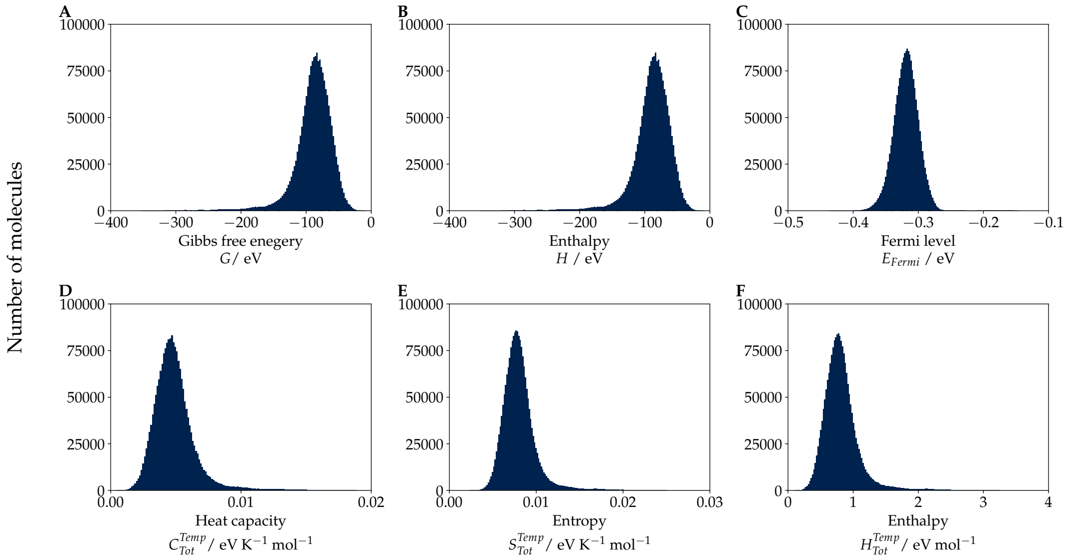

Figure S5: Thermodynamic property distribution for molecules in QMugs calculated on the GFN2-xTB level-of-theory. (**A**) Gibbs free energy G in eV. (**B**) Enthalpy H in eV. (**C**) Fermi level E_{Fermi} in eV. (**D**) Heat capacity C_{Tot}^{Temp} / eV K^{-1} mol^{-1}. (**E**) Entropy S_{Tot}^{Temp} in eV K^{-1} mol^{-1}. (**F**) Partition function enthalpy H_{Tot}^{Temp} / eV mol^{-1}.

8 Additional figures and tables

Table S1: Atom-type-specific atomic partial charge comparisons for the two levels of theory (GFN2-xTB, ωB97X-D/def2-SVP) for the QMugs database. Abbreviations: RMSE, root mean squared error; PCC, Pearson's correlation coefficient.

Atom type	Occurrence	RMSE	PCC
Hydrogen	49.0M	1.00×10^{-3}	0.913
Carbon	45.1M	0.0130	0.575
Nitrogen	7.28M	0.0264	0.124
Oxygen	6.08M	0.0188	0.274
Fluorine	1.14M	3.21×10^{-4}	0.868
Sulfur	729k	0.043	0.991
Chlorine	513k	6.39×10^{-3}	0.862
Bromine	82.5k	0.0153	0.831
Phosphorus	32.5k	0.139	0.872
Iodine	13.2k	0.0353	0.808

Table S2: Wiberg bond order comparisons for the two levels of theory (GFN2-xTB, ωB97X-D/def2-SVP) and the 15 most frequent pair-wise atomic covalent bonds in QMugs. Abbreviations: RMSE, root mean squared error; PCC, Pearson's correlation coefficient.

**Bond type**	**Occurrence**	**RMSE**	**PCC**
Carbon-Hydrogen	44.8M	1.30×10^{-3}	0.787
Carbon-Carbon	40.6M	6.83×10^{-4}	0.998
Carbon-Nitrogen	14.4M	0.0137	0.995
Nitrogen-Hydrogen	3.20M	2.86×10^{-3}	0.860
Carbon-Fluorine	1.14M	0.108	0.153
Carbon-Sulfur	1.13M	0.0223	0.977
Oxygen-Hydrogen	985k	0.0520	0.904
Carbon-Oxygen	683k	0.0972	0.998
Sulfur-Oxygen	618k	0.0853	0.941
Nitrogen-Nitrogen	538k	0.0278	0.997
Carbon-Chlorine	513k	0.0684	0.846
Nitrogen-Sulfur	249k	0.0139	0.964
Nitrogen-Oxygen	234k	0.110	0.997
Phosphorus-Oxygen	107k	0.0112	0.980
Carbon-Bromine	82.4k	0.0384	0.840

References

1. Ramakrishnan, R., Dral, P. O., Rupp, M. & Von Lilienfeld, O. A. Quantum chemistry structures and properties of 134 kilo molecules. *Sci. Data* **1**, 1–7 (2014).

2. Nakata, M. & Shimazaki, T. PubChemQC project: A large-scale first-principles electronic structure database for data-driven chemistry. *J. Chem. Inf. Model.* **57**, 1300–1308 (2017).

3. Smith, J. S., Isayev, O. & Roitberg, A. E. ANI-1, A data set of 20 million calculated off-equilibrium conformations for organic molecules. *Sci. Data* **4**, 170193 (2017).

4. Heller, S. R., McNaught, A., Pletnev, I., Stein, S. & Tchekhovskoi, D. InChI, the IUPAC international chemical identifier. *J. Cheminformatics* **7**, 1–34 (2015).

5. Weininger, D. SMILES, a chemical language and information system. 1. Introduction to methodology and encoding rules. *J. Chem. Inf. Comp. Sci.* **28**, 31–36 (1988).

6. O'Boyle, N. M. *et al.* Open Babel: An open chemical toolbox. *J. Cheminformatics* **3**, 1–14 (2011).

7. The Open Babel Package, version 3.1.1. Accessed September 2020. `http://openbabel.org`.

8. pyvenn: Venn diagrams for 2, 3, 4, 5, 6 sets (Accessed 07.05.2021). `https://github.com/LankyCyril/pyvenn`.

9. Mendez, D. *et al.* ChEMBL: Towards direct deposition of bioassay data. *Nucleic Acids Res.* **47**, D930–D940 (2019).

10. Oracle Corporation. MySQL. Accessed September 2020. `https://dev.mysql.com/`.

11. Landrum, G. RDKit: Open-source cheminformatics. Accessed September 2020. `http://www.rdkit.org`.

12. Tosco, P., Stiefl, N. & Landrum, G. Bringing the MMFF force field to the RDKit: Implementation and validation. *J. Cheminformatics* **6**, 37 (2014).

13. NIST Standard Reference Database 101. Computational chemistry comparison and benchmark database, Release 21, August 2020. Accessed September 2020.

14. Hagberg, A. A., Schult, D. A. & Swart, P. J. Exploring network structure, dynamics, and function using networkx. In Varoquaux, G., Vaught, T. & Millman, J. (eds.) *Proceedings of the 7th Python in Science Conference*, 11 – 15 (Pasadena, CA USA, 2008).

15. Bach, R. D. Ring strain energy in the cyclooctyl system. The effect of strain energy on [3 + 2] cycloaddition reactions with azides. *J. Am. Chem. Soc.* **131**, 5233–5243 (2009).

16. Grimme, S., Bannwarth, C. & Shushkov, P. A robust and accurate tight-binding quantum chemical method for structures, vibrational frequencies, and noncovalent interactions of large molecular systems parametrized for all spd-block elements (Z= 1–86). *J. Chem. Theory Comput.* **13**, 1989–2009 (2017).

17. Bannwarth, C., Ehlert, S. & Grimme, S. GFN2-xTB—An accurate and broadly parametrized self-consistent tight-binding quantum chemical method with multipole electrostatics and density-dependent dispersion contributions. *J. Chem. Theory Comput.* **15**, 1652–1671 (2019).

18. Grimme, S. Exploration of chemical compound, conformer, and reaction space with meta-dynamics simulations based on tight-binding quantum chemical calculations. *J. Chem. Theory Comput.* **15**, 2847–2862 (2019).

19. Bannwarth, C. *et al.* Extended tight-binding quantum chemistry methods. *Wiley Interdiscip. Rev. Comput. Mol. Sci.* e01493 (2020).

20. Born, M. & Oppenheimer, R. Zur quantentheorie der molekeln. *Annalen der physik* **389**, 457–484 (1927).
